# Supplementary material for: Predicting Immunotherapy Outcomes in Glioblastoma Patients through Machine Learning
Source: Cancers (Basel). 2024 Jan 18;16(2):408. doi: 10.3390/cancers16020408 (PMC10813889; doi:10.3390/cancers16020408)
Supplement: Supplementary file 1 [file cancers-16-00408-s001.zip › cancers-2814539-supplementary.pdf]

Supplementary Figures

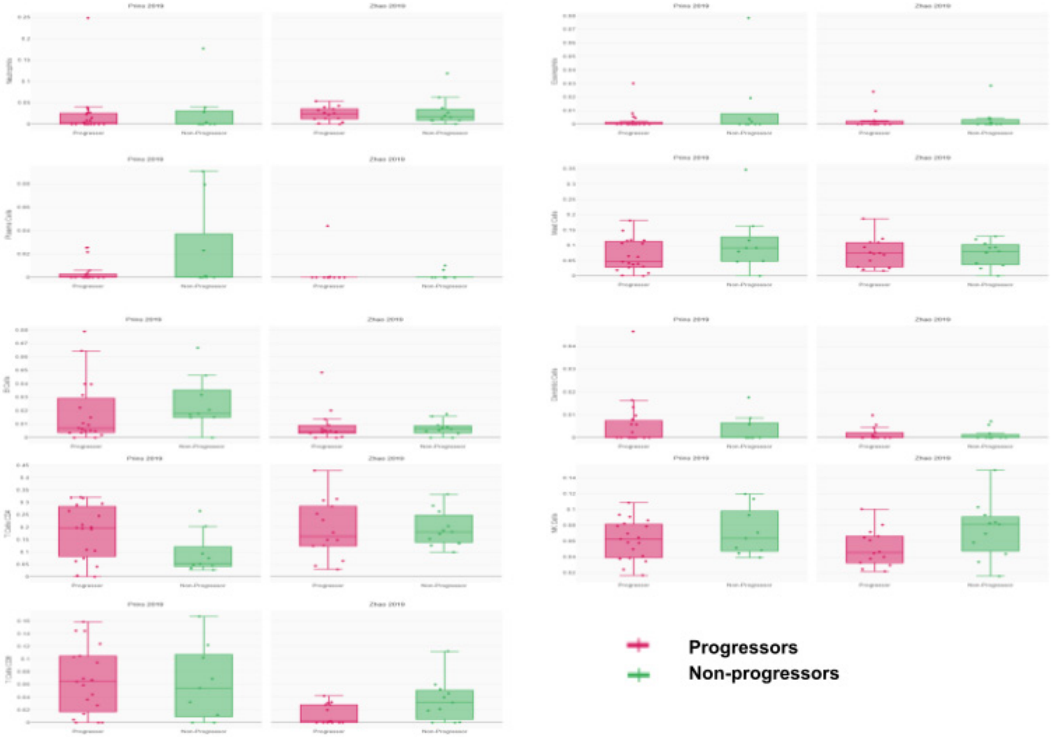

Supplementary Figure S1. Immune response in glioblastoma patients according to progression following PD1 blockade [2,5].
62 patients received anti-PD-1 following glioblastoma. n = 28 for Nivolumab (Zhao) and n = 34 for Pembrolizumab (Prins). Immune response is measured using CRI iAtlas. $p < 0.05$, Wilcoxon t-test.

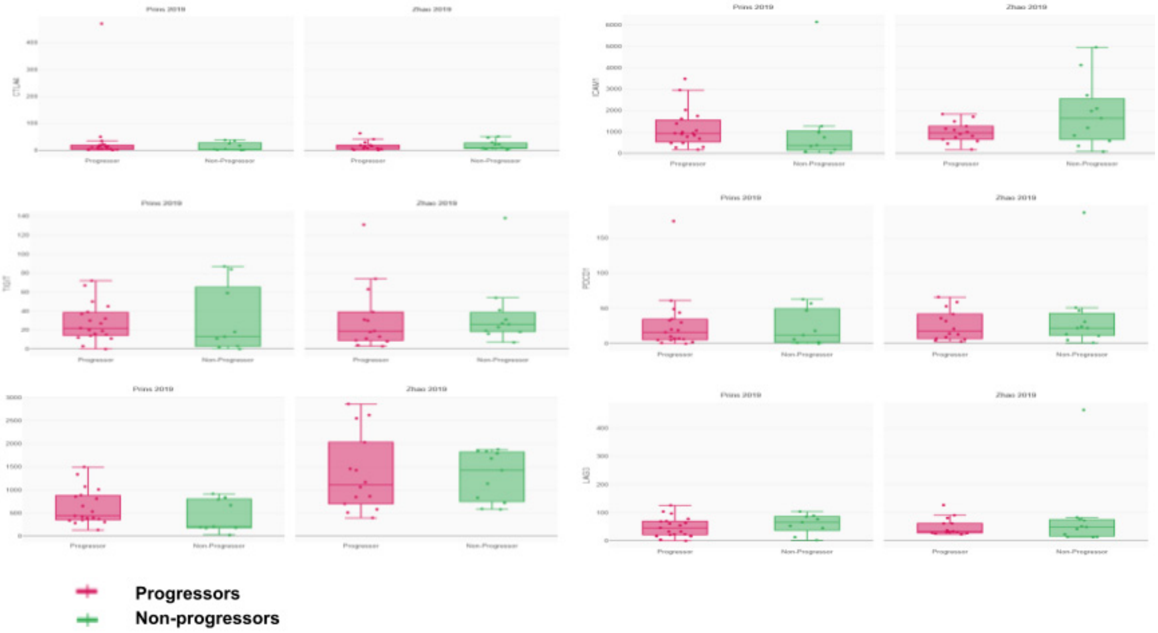

Supplementary Figure S2. Immunomodulatory molecule expression in glioblastoma patients according to progression following PD1 blockade [2,5].
62 patients received anti-PD-1 following glioblastoma. n=28 for Nivolumab (Zhao) and n = 34 for Pembrolizumab (Prins). Immunomodulatory molecule expression is measured using CRI iAtlas. $p < 0.05$, Wilcoxon t-test.
